# Clinical leadership in nursing homes: A qualitative study of healthcare professionals' perspectives on concept and characteristics

**DOI:** 10.1002/nop2.2166

**Published:** 2024-06-07

**Authors:** Sabrina Nachtergaele, Nele De Roo, Jolien Allart, Patricia De Vriendt, Mieke Embo, Elise Cornelis

**Affiliations:** ^1^ Research and Innovation Centre Health and Care Artevelde University of Applied Sciences Ghent Belgium; ^2^ Nursing Department Artevelde University of Applied Sciences Ghent Belgium; ^3^ Occupational Therapy Department Artevelde University of Applied Sciences Ghent Belgium; ^4^ Frailty in Ageing (FRIA) Research Group, Mental Health and Wellbeing Research Group (MENT), Gerontology Department Vrije Universiteit Brussel Jette (Brussels) Belgium; ^5^ Department of Rehabilitation Sciences, Occupational Therapy Research Group, Faculty of Medicine and Health Sciences Ghent University Ghent Belgium; ^6^ Department of Educational Studies, Faculty of Psychology and Educational Sciences Ghent University Ghent Belgium

**Keywords:** clinical leadership, competencies, elder care, nursing home, person‐centred

## Abstract

**Aim(s):**

To conceptualise and identify characteristics of clinical leadership in the nursing home setting.

**Design:**

A qualitative study using semi‐structured focus group interviews and a thematic analysis.

**Methods:**

Five semi‐structured focus group interviews were conducted with 41 healthcare professionals from nursing and other healthcare disciplines working in nursing homes (such as nurse assistants, licensed practical nurses, registered nurses (RNs), occupational therapists, recreational therapists, psychologists and gerontologists). Qualitative thematic content analysis of the gathered data was done.

**Results:**

Clinical leaders in nursing homes can be defined as passionate healthcare professionals providing person‐centred care with strong communication skills. They are clinical experts in their field and motivated to engage in lifelong learning. They are team players with informal leadership skills. They are visionary, committed, resilient and responsive. Awareness of the definition and the main characteristics of clinical leadership is necessary to facilitate the identification, support and development of healthcare professionals. Focussing on the development of competencies, training courses and monitoring and assessment methods is necessary to improve the evidence of clinical leadership in nursing homes.

## 1 INTRODUCTION

Globally, healthcare systems are facing tremendous challenges to ensure that they are ready to make the most of the fast pace of population ageing (World Health Organisation, 2021). In elderly care, there is growing consensus that a skilled nursing and care workforce is important to ensure higher healthcare professional‐related outcomes and positive outcomes for older persons (Backman et al., 2018; Boamah, 2019; Cummings et al., 2018). The crucial role of clinical leaders in healthcare, particularly in elderly care such as in nursing homes, became abundantly clear since the COVID‐19 pandemic exposed the weaknesses in global healthcare systems. Current evidence suggests that COVID‐19 disproportionately affected older adults and persons with chronic health conditions, leading to higher attack rates and more severe adverse outcomes among them (Alves et al., 2021). A confluence of factors in the 21st century makes leadership capacity particularly relevant to practice in nursing homes (Poels et al., 2020).

Clinical leadership is an informal type of leadership in the healthcare setting. Harper (1995) was one of the first researchers to use this concept in nursing care. He defined clinical leaders as nurses with clinical expertise in specialised domains who use interpersonal communication skills to support colleagues to deliver high‐quality patient care. Other researchers defined nurse clinical leaders as nurses who provide day‐to‐day care, act as role models, influence, motivate and inspire others with their values and beliefs to improve care, although they have no formal leadership role (Stanley & Stanley, 2018). The word clinical refers to the history of clinical leadership that was mainly studied among nurses in the hospital setting. Research explains the concept and its features and explores the positive impact of clinical leadership on nurses' quality of care and job satisfaction (Enghiad et al., 2022). During the last decade, care changed with a growing emphasis on multidisciplinary care and new profiles of healthcare professionals emerged. In this new context, Stanley and Stanley (2018) stated that the concept of clinical leadership needed to broaden to all healthcare professionals in several healthcare settings, such as nursing homes.

Nursing homes to date, face numerous challenges in providing quality of care and quality of life. Firstly, competent healthcare professionals in nursing homes are required to provide qualitative care in a complex and stressful working environment. This is necessary because older residents are a vulnerable population at higher risk of morbidity and mortality (Dosa et al., 2020; Ouslander & Grabowski, 2020). The residents' advanced age causes more frequent and complex chronic disease patterns, an increased risk of falling and severely compromised physical, cognitive and immune system functions that place them at the highest risk of life‐threatening infections (Temkin‐Greener et al., 2020). Secondly, staffing issues compromise patient safety and care quality. Nursing homes have low nurse staffing competency levels despite the existence of strong evidence and widespread agreement that higher nurse staffing levels, particularly higher RN levels, improve safety of care and resident outcomes such as fewer medication errors and lower mortality rates (Temkin‐Greener et al., 2020). Next, staff shortage and an excessive workload lead to a field of practice with relatively high rates of nursing turnover. Because leadership has been linked to better quality measures and health outcomes (Enghiad et al., 2022) and because it has been linked to lower rates of nursing turnover (Enghiad et al., 2022; Fiset et al., 2017), it is crucial to develop clinical leadership in practice.

Thirdly, it is challenging to develop clinical leadership competencies in the setting of nursing homes, since there are no competency frameworks for clinical leaders in nursing homes and the research on the characteristics of clinical leaders in hospitals is inconsistent and complex (Stanley & Stanley, 2018). Mannix et al. in Stanley and Stanley (2018) found in their systematic review three categories of characteristics: focus on ‘clinical work’, ‘teamwork’ and ‘personal qualities’. The primary characteristic of clinical leadership has been recognised as clinical competency (Stanley & Stanley, 2018). Effective communication, support, focus on values and beliefs, approachability, being a decision‐maker, visible in practice, serving as a role model, focus on excellence and providing quality care are other qualities of a clinical leader (Stanley & Stanley, 2018).

Despite the workforce challenges, healthcare professionals in nursing homes frequently experience their work as meaningful (Eriksson et al., 2023; Rajamohan et al., 2019). Many best practices in nursing homes around the world show that healthcare professionals give their all to improve the lives of residents. According to Plaku‐Alakbarova et al. (2018), there are positive correlations between the job satisfaction of healthcare professionals and the general satisfaction of residents. Especially when healthcare professionals are working in a person‐centred way, they can do a little extra for residents which is what they thrive on when working in nursing homes (Rajamohan et al., 2019; Vassbø et al., 2019). Additionally, some healthcare professionals in nursing homes serve as role models and their behaviour is considered as a standard of excellence to strive for (Mrayyan et al., 2023; Stanley & Stanley, 2018). Despite lacking formal authority, they can influence, motivate and inspire staff to provide high‐quality care and person‐centred guidance. They are clinical leaders who can improve the standards of care, outcomes and satisfaction for residents (Boamah, 2019; Enghiad et al., 2022) and provide beneficial results for healthcare professionals such as improved job satisfaction, intention to stay and well‐being (Backman et al., 2018; Boamah, 2019; Enghiad et al., 2022).

Nursing homes might need more clinical leaders in the future because of the ageing population. Given the beneficial effects of clinical leadership on patients and staff in hospitals and the above‐mentioned challenges in nursing homes, it is time to research the concept and characteristics in this context. Although the concept of clinical leadership has already been extensively researched by nurses in hospitals, there is no clear definition of clinical leadership within healthcare professionals in nursing homes (Stanley & Stanley, 2018). Research is urgently needed to conceptualise clinical leadership in nursing homes and to grasp the characteristics needed to become impactful clinical leaders in this setting. This qualitative study aims to explore the concept of clinical leadership and to identify the characteristics of clinical leaders in nursing homes. We will answer the following research questions by conducting a qualitative focus‐group study with healthcare professionals in nursing homes.

Research question 1 (RQ1): How do healthcare professionals in nursing homes define clinical leadership?
Research question 2 (RQ2): How do healthcare professionals in nursing homes identify the characteristics required of clinical leaders in these facilities?

## 2 METHODS

### 2.1 Design

We conducted a qualitative study in Flanders (Dutch‐speaking Region in Belgium) using semi‐structured focus group interviews and a thematic analysis. This study complies with the Consolidated Criteria for Reporting Qualitative Research (COREQ) (Booth et al., 2014).

### 2.2 Context

Belgium has three levels of government, the federal, regional and linguistic community divisions, with each having different responsibilities. Three distinct regional administrations are responsible for the organisation of nursing homes (Flanders, Walloon and Brussels). Nursing homes can range in size, from those with fewer than 50 beds to those with more than 150 beds, they can be found in either rural or urban areas and they have various funding models.

### 2.3 Participants

All licensed nursing homes in East (*n* = 198) and West Flanders (*n* = 161) were contacted, using the registered mailing address of the government website of nursing homes (https://www.zorg‐en‐gezondheid.be/per‐domein/ouderenzorg/woonzorgcentra). No data was available on the number of employed healthcare professionals in licensed nursing homes. To encourage healthcare professionals to take part in a focus group interview, the main investigator (SN) sends emails outlining the objectives of the study. Dutch‐speaking healthcare professionals employed in nursing homes in East and West Flanders were included. Healthcare professionals providing direct care in nursing homes were included to participate in the focus groups. Nursing home staff who did not directly provide care to residents, such as kitchen workers and housekeeping assistants, were not included. Interested healthcare professionals were asked to send an email to SN to organise the focus group interviews. Three focus groups were set up with a total of 19 willing participants who responded by email. In a second round, in order to increase the diversity of participants, two additional focus groups were conducted with 22 healthcare professionals. In total, 41 healthcare professionals including nurse assistants, licensed practical nurses, RNs, occupational therapists, recreational therapists, psychologists and gerontologists were enlisted to take part in five focus groups (Table 1). The European Qualification Framework was used to clarify the nursing profession (The Council of the European Union, 2017) (Table 4). In this, 25 healthcare professionals (61%) with no formal leadership role and 16 healthcare professionals (39%) with a formal leadership role (e.g. nurse leaders, nursing home managers, etc.) participated in the focus groups. These healthcare professionals varied greatly in age, amount of work experience and educational degree. The nursing home where they worked varied in size, financial model and location.

**TABLE 1:** Demographic characteristics of the participants of the focus groups.

Characteristics	Focus group 1 (*n* = 5)	Focus group 2 (*n* = 5)	Focus group 3 (*n* = 9)	Focus group 4 (*n* = 9)	Focus group 5 (*n* = 13)	Total (*n* = 41)
Duration of the focus group	1 h 43 min	1 h 44 min	1 h 46 min	53 min	1 h 18 min	7 h 24 min
Sex						
Men	0	0	8	0	4	12 (29.3%)
Women	5	5	1	9	9	29 (70.7%)
Age (years)						
<35	2	4	2	2	8	18 (43.8%)
35–44	1	0	4	3	2	10 (29.5%)
45–54	0	0	2	1	1	4 (9.7%)
>55	1	1	2	1	2	7 (17.0%)
Amount of work experience (years)						
<3	0	2	0	1	3	6 (14.6%)
3–5	0	1	1	1	3	6 (14.6%)
6–10	2	0	1	0	3	6 (14.6%)
>10	3	2	7	7	4	23 (56.2%)
Educational degree (EQF)						
Post‐secondary education (EQF 4)	0	1	1	4	10	16 (39.0%)
Graduate education (EQF 5)	0	0	0	2	1	3 (7.3%)
Bachelor's degree (EQF 6)	3	2	6	2	1	14 (34.1%)
Master's degree (EQF 7)	2	2	2	1	1	8 (19.6%)
Nursing home size (bed capacity, *n*)						
<50	0	0	0	0	0	0 (0%)
50–100	0	0	1	0	0	1 (2.4%)
100–150	0	1	4	0	0	5 (12.2%)
>150	5	4	4	9	13	35 (85.4%)
Financial model						
Non‐profit	2	0	2	0	13	17 (41.5%)
Private nursing home	2	5	3	9	0	19 (46.3%)
Public nursing home	1	0	4	0	0	5 (12.2%)
Location						
Urban	4	5	3	9	13	34 (83.0%)
Rural	1	0	6	0	0	7 (17.0%)

Abbreviation: EQF, European Qualifications Framework (The Council of the European Union, 2017).

### 2.4 Data‐collection

To facilitate discussion among healthcare professionals about clinical leadership in nursing homes, we used semi‐structured focus groups. Focus groups fit within a constructivist paradigm and are well suited for exploring the circumstances through which participants construct meaning, making this an appropriate tool for exploring the context surrounding healthcare professionals' experiences with clinical leadership in nursing homes (Sawatsky et al., 2019).

Before the start of the focus group interviews, two researchers (SN and EC) developed an interview guide during several brainstorming sessions (Table 3). To get a rich view of participants' experiences, it was decided to only use open‐ended and non‐directive questions (Sawatsky et al., 2019).

The focus groups were organised between November 2019 and January 2020. The first three focus groups were organised at Artevelde University of Applied Sciences (Ghent) and the other two focus groups were organised at one of the participating nursing homes (Ghent). The duration of the interviews varied between one and a half and two and a half hours. Each focus group was led by the main investigator (SN) who was trained in qualitative research techniques. The first focus group was organised with a senior researcher (EC) to guarantee the quality of the procedure. Each focus group followed a similar twofold procedure. First, after obtaining informed consent, the semi‐structured interview guide was used to explore the healthcare professionals' point of view about the concept (RQ1) and characteristics (RQ2) related to clinical leadership in nursing homes. Participants were first asked to think of a colleague whom they look up to in their role as healthcare professionals. Secondly, they were asked to give a description of this colleague's personal characteristics, defined as typical or noticeable qualities of someone (Cambridge Dictionary, n.d.). Finally, printed cards with characteristics of a clinical leader in hospital and primary care as stated by Mannix et al. (2013) were laid out on a table. The participants were invited to choose and discuss relevant clinical leadership characteristics of healthcare professionals in nursing homes.

### 2.5 Data‐analysis

The focus group interviews were audio recorded, transcribed and anonymised, meaning that the main researchers (SN, EC) removed any identifying information. Focus groups were manually coded in an Excel file. A qualitative thematic content analysis was chosen in which all data were deconstructed and reorganised according to similar content (Hewitt‐Taylor, 2001). First, the two researchers (EC and SN) independently and inductively analysed the transcribed focus groups line‐by‐line. They used open coding with in vivo codes, meaning that codes were formed based on the words used by the participants to ensure an open‐minded approach. Subsequently, they examined and discussed relationships between codes, using the constant comparison and clustering method. A third researcher (PD) provided advice throughout the process of thematic analysis of the data. This resulted in an in‐depth analysis of the data that allowed the naive understanding of the data to evolve into a comprehensive understanding with all data reunited into meaningful themes (Table 2). Data saturation was achieved after the analysis of the fourth focus group. Data from the fifth focus group was also included in the data collection. Organising additional focus groups was not necessary. To establish the trustworthiness of the study findings, member checking was performed by presenting summarised thematic analyses to three participants who corroborated the author's interpretations of the data (Sawatsky et al., 2019). This process did not identify any need for further analysis or data revision.

**TABLE 2:** Results of the data analyses.

In vivo codes (Dutch)	Category (Dutch/English)	Theme (Dutch/English)
Doet job vanuit een passie; dynamisch; dynamisch en gedreven zijn; enthousiasme doorgeven; enthousiast; enthousiast, dynamisch, gedreven; gedreven; gedreven, dynamisch, enthousiast; geeft kracht; goesting hebben; groot hart voor bewoners, lief met bewoners; hart voor de bewoners; heeft een hart voor het werk; legt eigenheid in job; liefde voor de job hebben; niet gewoon deelnemen; optimistisch; overtuiging voor job; passie voor de job; vanuit het hart doen; werken met hem/haar is fijn	Bezieling (passion)	Bevlogenheid (committed)
Bezit een positieve attitude t.a.v. beroep; goed humeur, opgewekt, vrolijk; lacht veel, bezig vol enthousiasme, met een glimlach; positief ingesteld; positief ingesteld, positieve attitude; positief, positiviteit; positiviteit	Positief ingesteld (positive attitude)	
Graag voor WZC werken; interesse in werkdomein; inzet naar bewoners en collega's; inzetten voor patiëntgerichte zorg; met hart en nieren in WZC werken; neemt uitdagingen aan; willen er echt voor gaan; zichzelf inzetten	Toewijding (dedication)	
Ambassadeurs van goede zorg; stralen iets uit in hun vakgebied	Uitdragen (ambassadeur)	
Aandacht geven; aandacht voor de bewoner; betrokken communicatie; communicatief vaardig: kan aanpassen aan verschillende situaties en contexten; extra informatie geven; geeft aandacht aan anderen; gerichte communicatie; herkenning bieden aan collega's; kan omgaan met verschillende soorten personen (familie, …); neutraal; sociaal vaardig; vermogen aan te passen aan verschillende contexten	Adaptief (adaptive)	Effectieve communicatie (effective communicator)
Communicatief; communicatief sterk; communicator; effectief communiceren; efficiënte communicatie; heldere besluitvorming	Bondig en helder (brief and clear)	
anderen durven aanspreken; collega's attent maken; conflicthanteerder, probleemoplosser; feedback geven en vragen; feedback kunnen ontvangen; geeft opbouwende feedback; geven van feedback; kan betrokken confronteren; onderhandelen; onderhandelend; oplossingen bespreken in team; positief bekrachtigen; positieve feedback; positieve feedback geven; vraagt en geeft feedback, durft minpunten te benoemen, biedt erkenning	Feedback geven en ontvangen (give and receive feedback)	
Gaat in gesprek; geduldig; geeft raad en advies; in communicatie gaan; kan betekenisvolle gesprekken voeren; openstaan om tips te geven	In dialoog gaan (engage)	
actief luisteren; communicatief, actief luisteren; heeft een luisterend oor; luisterend oor, actief luisteren; open staan om te luisteren; rust uitstralen; staat open om te luisteren en te helpen; tijd nemen	Luistervaardigheden (listening skills)	
bekrachtigt positief; enthousiast; inspirerend; kan rust geven; motiveert; straalt rust uit	Overtuigingskracht (persuasiveness)	
Flexibel; flexibel, kan zich aanpassen; flexibiliteit; goed omgaan met verandering; goed omgaan met veranderingen; neemt tijd; routines doorbreken	Aanpassingsvermogen (adaptability)	Flexibiliteit (resilient)
Kan goed omgaan met werkdruk; stressbestendig	Stressbestendigheid (stress resistance)	
Breed en ruimdenkend zijn; ruimdenkend	Ruimdenkend (open‐minded)	
Aandacht voor elkaar mening; creatief; dingen op eigen manier doen; durf; extra aandacht geven; onpartijdig, neutraal; tijd nemen om er te zijn; tijd nemen voor anderen (bewoners en collega's); tijd nemen voor bewoners; verschillende insteken aanvaarden	Variabiliteit (variability)	
Gaat niet mee in een negatieve spiraal; kan ombuigen naar een positief verhaal; relativeringsvermogen; vaardigheid om oplossingen van problemen te zien; vermogen om te relativeren	Veerkracht (resilience)	
Anderen helpen; bereidwillig t.a.v. bewoners; bereidwillig, behulpzaam; biedt hulp; collega's helpen; elkaar helpen; extra taak opnemen; functie‐overschrijdend werken; gemeenschappelijke taken opnemen, niet enkel eigen takenpakket; iemand die je komt helpen; iets extra doen; iets overnemen; inspringen; méér doen; meer doen dan er gevraagd wordt; méér kunnen betekenen; springt in, helpt als eerste; stelt het belang van anderen voorop zonder zichzelf te verliezen	Welwillend (benevolent)	
Bewonersgericht; empoweren t.a.v. bewoner; geheel zien; het geheel zien, het totaalplaatje; iets naar een hoger niveau tillen; kan hoofd‐ en bijzaken onderscheiden; meer dan zorg; patiëntgericht; rapporteert het totaalplaatje; rapporteren over binnenkant van mensen; stelt bewoner centraal, aandacht ligt op de bewoner, patiëntgericht; totaalzorg geven; uitleg geven aan families; zet zich in voor gerichte zorg; zorg en welzijn van bewoners steeds in gedachten houden	Bewonersgericht (person‐centred)	Klinische expertise (clinical expert)
Bekwaam; deelt expertise; deskundigheid; ervan bijleren; faciliteren goede praktijk en zorg; faciliteren van goede praktijk en zorg, ondersteunend; georganiseerd; gestructureerd werken; goede, warme zorg leveren; heeft veel kennis, deskundig, expert; interesse in werkdomein, goede algemene kennis; kan georganiseerd werken; kan je om raad en advies vragen; kennen van de job; kennis; kennis delen; kwaliteitsvol werk afleveren; kwaliteitsvolle zorg leveren; op de hoogte van alles; respecteren van professionele gedragscodes; rustig, gestructureerd, systematisch werken; uitgebreide kennis, knowhow, professionaliteit; veel kennis; vermogen om snel te denken; vertrekt vanuit een goede, warme zorg; werkt georganiseerd; werkt kwaliteitsvol; weten waar ze mee bezig zijn	Deskundig (expert)	
Enthousiast om bij te leren; grenzen kennen; leerproces bevorderen; nieuwste ontwikkelingen volgen; opleiding; probeert zoveel mogelijk informatie op te zoeken; volgt nieuwste ontwikkelingen; vormingen geven; zich ergens in bekwamen	Levenslang leren (lifelong learning)	
Alert; analyseren; heldere besluitvorming; hoofdzaken van de bijzaken kan onderscheiden; kritisch denken; kritische denker; kritische denker, kritisch zijn; redeneren; verbanden leggen	Professioneel redeneren (critical thinking)	
Anciënniteit: diegene die het langst in dienst zijn; ervaring; heeft veel ervaring	Werkervaring (work experience)	
Empathisch	Empathisch (empathetic)	Mensgericht (person‐centred)
Hartelijk, zorgzaam, vriendelijk, warm; stralen een soort warmte uit; vriendelijk; warmte; zorgzaam	Hartelijk (cordial)	
Authenticiteit; echt; geeft vertrouwen; integer; integriteit; met een natuurlijke flair; naturel; stralen vertrouwen uit; vertrouwen geven, opbouwen, krijgen; zelfvertrouwen; zichzelf zijn	Integriteit (integrity)	
Altruïsme; goed willen doen; heeft het beste voor met de bewoners; heeft intentie om goed te doen; heeft intentie om goed te willen doen; intentioneel; niet oordelen; rechtvaardig; respect; respect uitdragen; vraagt hoe het gaat; wil het doen; wil het goed doen; zonder te oordelen	Intentie om het goed te doen (intention to do well)	
Bereikbaar t.a.v. bewoners; bescheiden; betrokken; betrokken bij bewoners; betrokken bij familie; betrokkenheid; biedt een gevoel van vertrouwen; biedt houvast; er zijn voor anderen; iemand bij wie je terecht kan; open; open en toegankelijk	Nabijheid (proximity)	
Focus op menselijke relaties; interactief met bewoners; promoot betrokkenheid; relaties waardevol vinden; relaties waardevol vinden: samenwerken, klaar staat voor begeleiding, ondersteund anderen, denkt aan anderen	Relaties waardevol vinden (value relationships)	
Heeft een vermogen om signalen op te pikken; kan een probleem aanvoelen; signalen van anderen oppikken	Sensitiviteit (sensitivity)	
Anderen enthousiasmeren en motiveren, mensen op sleeptouw nemen; anderen stimuleren en motiveren; beïnvloeden; elkaar meekrijgen in team; empoweren t.a.v. collega's; helpt het team groeien; kan een team mobiliseren; laat anderen zich ontwikkelen; laat anderen zichzelf ontwikkelen; mensen die je optrekken; mobiliseren van het team; motivatie, motiverend; motivator; motiveren; motiveren van collega's; motiveren, motivator; motiverend; probeert mensen mee te trekken; zorgt dat het team vooruit gaat	Aanzetten tot (incite)	Teamvaardig (team based worker)
Aangenaam om mee samen te werken; aanvullend in team; collegiaal; collegialiteit; de lijm; erkennen van waarden en normen van collega's; goed omgaan met het team; herkennen eigen sterktes en zwaktes; herkennen van waarden en normen van collega's; houden de boel bijeen, zijn de lijm; kan een groep aanvoelen; ondersteunen; ondersteunend; ontwikkelen van samenwerking; ontwikkelen van samenwerkingsverbanden; opnemen voor de groep; respect voor collega's; respect voor het MDT; samenwerken; teamspeler; teamspeler, teamplayer; teamvaardigheden; team verbinden; vieren van prestaties van collega's	Collegiaal (collegial)	
Geeft leiding; gerespecteerd; iemand waar je naar opkijkt; kan aansturen; krijgen mandaat om complimenten te geven; krijgen mandaat om te zeggen als het minder goed gaat; mee gaan sturen of gaan aansporen; nieuwe benaderingen aansturen; richting geven; rol hebben in de organisatie van de afdeling; stuurt aan; stuurt in verschillende situaties; team kijkt naar die persoon; wordt gedragen in het team; zeggingskracht hebben	Informele leiderschapsvaardigheden (informal leadership skills)	
Bereikbaar; bereikbaarheid; erop kunnen rekenen; kan je op rekenen als je iets nodig hebt; klaar staan voor anderen; open, toegankelijk; toegankelijk; toegankelijk, bereikbaar	Toegankelijk (accessible)	
Anticipeert op probleem en werkt eraan die te voorkomen; beslissingnemer; betrokken, verantwoordelijkheidszin; collega's vertegenwoordigen; durft situaties aan te pakken; engagement; gedeelde verantwoordelijkheid; gedeelde zorg bieden; iemand die bal aan rollen kan brengen; initiatiefnemer; mogelijk maken; neemt verantwoordelijkheden op; neemt verantwoordelijkheid; neemt verantwoordelijkheid op; ondernemend; opmerkingen interdisciplinair delen; probleemoplossend; probleemoplosser; verantwoordelijk; verantwoordelijkheid; verantwoordelijkheid dragen; verantwoordelijkheid hebben	Daadkracht (decisiveness)	Verantwoordelijkheidszin (responsive)
Betrouwbaar; consequent; geëngageerd, plichtsbewust; heeft oog voor detail, aan kleine zaken belang hechten; job serieus nemen; nauwkeurig; oplettend; opmerkzaam; plichtsbewust; vertrouwelijk; vertrouwelijkheid, vertrouwen uitstralen; vertrouwen hebben; vertrouwen, betrouwbaar; zelfkennis	Zorgvuldig (careful)	
Denkt mee in het geheel; dragen de visie uit; engagement; geëngageerd; loyaal; meedenken; respect voor brede organisatie	Betrokkenheid (involvement)	Visiedrager (visionary)
Bewaakt het ‘pad’ of de ‘lijn’ dat bewandeld wordt, heeft visie, weet waar je naartoe wil; doelbewust; doelgericht; gemeenschappelijk doel voor ogen hebben; wil vooruit	Doelbewust (purposively)	
Denkt mee, denkt niet alleen binnen de lijnen; krijgt dingen gedaan door visie die men uitstraalt; sterke visie; visie op de toekomst	Visionair (visionary)	

**TABLE 3:** Interview guide applied during the semi‐structured focus groups.

*Opening question*
Consider a colleague whom you ‘look up to’ in his or her role as a health care provider. This may be someone who acts as a role model for you and perhaps acts as a clinical leader in your department
*Questions to define the concept of clinical leadership in nursing homes*
Describe the colleague you have taken to mind Why is he or she a role model for you? What makes you look up to this colleague? Specifically name what he or she does. What makes him or her do this?
*Questions to define the characteristics linked to the concept of clinical leadership*
On this table are all the possible attributes of a ‘clinical leader’. Pick out the characteristics that for you fit the colleague you just described as a role model Explain why you have chosen these characteristics Does everyone arrive at the same characteristics? Are there any differences between the chosen characteristics?
*Closing questions*
If you had to arrive at one description of a clinical leader based on everyone's story: What would this description look like? What characteristics would be in this description then?

*Note*: All the focus groups were conducted in the Dutch language; the original interview guide was also written in Dutch. For the interview guide, presented in the supplement the original interview guide was translated into English.

**TABLE 4:** Overview of participants' healthcare professions, their educational degree and corresponding European Qualification Framework level (The Council of the European Union, 2017).

Healthcare profession (*n* = 41)	Educational degree	EQF level
Nurse assistants (*n* = 16)	Post‐secondary education	EQF4
Licensed practical nurses/recreational therapists (*n* = 3)	Graduate education	EQF5
Registered nurses/occupational therapist (*n* = 14)	Bachelor's degree	EQF6
Psychologists/gerontologists (*n* = 8)	Master's degree	EQF7

### 2.6 Ethics

The study was conducted in full accordance with the 1975 Declaration of Helsinki and the revised version of 1983 (Carlson et al., 2004).

## 3 RESULTS

### 3.1 Perspectives on the concept of clinical leadership

The analysis of the responses to the first interview question, ‘think of a colleague, a role model and a healthcare professional, who provides direct patient care, you look up to’ revealed that every participant could name one or several colleague(s) they considered as role models or clinical leaders. Participants and their role models' backgrounds in the workplace varied. They all emphasised how important their role models were for the multidisciplinary team. The named characteristics are recognised by healthcare professionals. Most participants had not just one but several role models in mind during the focus group discussions. Describing these multiple role models revealed several characteristics that encompassed clinical leadership. Several participants referred to clinical leaders possessing *informal leadership skills*. It was hard for participants to find perceptions that provided more information about the definition of clinical leadership, yet the named characteristics were recognised by healthcare professionals.

### 3.2 Perspectives on the competencies of a clinical leader

**FIGURE 1:**
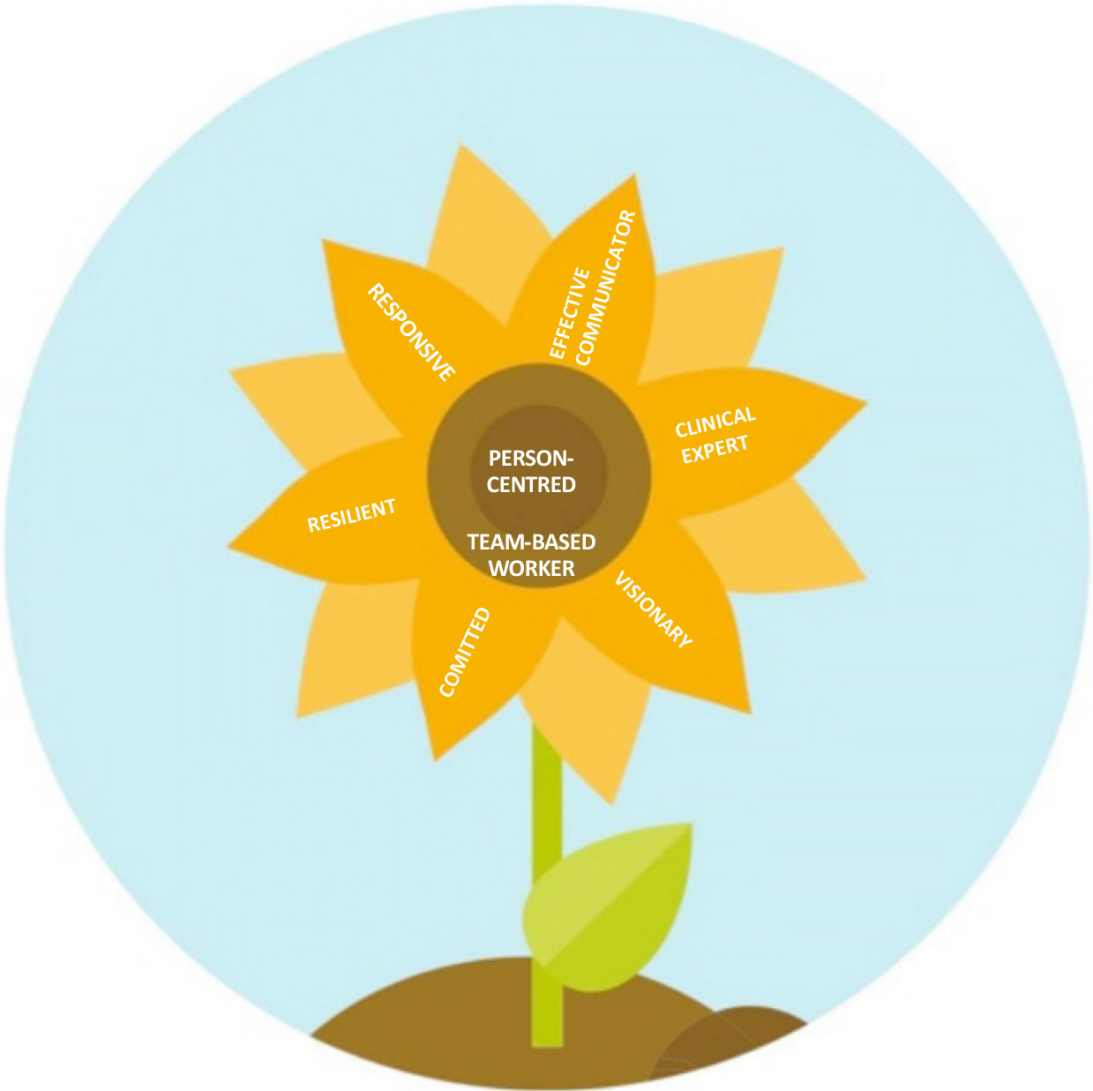
Visual of the clinical leadership model of care.

The analysis of the clinical leaders' personal characteristics in nursing homes was grouped into eight themes: (1) person‐centred, (2) effective communicator, (3) clinical expert, (4) team‐based worker, (5) visionary, (6) committed, (7) resilient and (8) responsive. A figure was designed to present the model of clinical leadership in nursing homes (Figure 1).

#### 3.2.1 Person‐centred

All participants perceived that clinical leaders put the resident at the centre of care. Clinical leaders are characterised by their *empathy* and *integrity*. They are authentic, sincere and real. They behave naturally and confidently, and because of their natural flair, they win the trust of residents, their families and colleagues. They are caring, behave warmly and friendly and show *cordiality*. They perform their daily tasks with the *intention of doing well*. They radiate *proximity*, are open and accessible. These healthcare professionals *value relationships* and spontaneously show a great commitment to the life and well‐being of others, they care for others and promote mutual involvement between healthcare professionals and residents. A clinical leader has a certain *sensitivity* which enables the detection of warning signals.

Clinical leaders pay attention to the aspect of care, as well as to the aspect of housing and living. (Healthcare professional with no formal leadership role, FG4)

#### 3.2.2 Effective communicator

Clinical leaders have strong communication skills and communicate *briefly and clearly* towards residents, family and colleagues. They are patient and radiate a certain calmness, which allows them to *engage* and take time for a meaningful conversation. In addition, they possess adequate *listening skills*: listening actively, being attentive, communicating concisely and giving advice. These healthcare professionals use their communication *adaptively* and pace their communication style in different situations, contexts and target groups. They deal with neutrality and detect where it is necessary to give additional information and attention to others.

Clinical leaders can easily *give and receive feedback* and use their communication skills appropriately to resolve conflicts. They do not only provide constructive feedback; they also offer recognition by giving compliments. They possess *persuasiveness*, actively negotiate and do not hesitate to share their comments.

Briefing with her is always very efficient! She can say a lot with few words, all information is presented succinctly but correctly. (Healthcare professional with a formal leadership role, FG1)

#### 3.2.3 Clinical expert

Clinical leaders are *experts* who have extensive knowledge and interest within their field of work, often accompanied by several years of *work experience*. They know their job and are up‐to‐date. Moreover, they are described by colleagues as the ones you can learn from. They are willing to share their expertise and knowledge and colleagues can always ask them for advice. Clinical leaders always work in an organised and structured manner and strive to give residents and their families good, warm and qualitative care. They are experts in providing *person‐centred* care for the vulnerable older population in a nursing home (e.g. persons with dementia) and are successful in getting a total picture of the resident and their surroundings. In other words, they go above and beyond basic caregiving to provide higher‐quality care.

Clinical leaders are dedicated to *lifelong learning*, follow up on the evidence within their field of work, and are enthusiastic about learning and doing training courses. They are strong in *critical thinking*, alert and they consider situations carefully before acting. They provide high‐quality care within professional boundaries.

Even though I've been in healthcare for so long, I still have colleagues from whom I say: damn, I can learn a lot from them. (Healthcare professional with no formal leadership role, FG5)

#### 3.2.4 Team‐based worker

Clinical leaders *incite* their colleagues. They ensure that the team is mobilised and moving forward.

In addition, clinical leaders can stimulate, motivate, influence and encourage others to act. They are *collegial* and respectful of their colleagues across the entire multidisciplinary team. They collaborate well within the team and are committed to fostering this collaboration. They create a connection between different team members and stand up for the team when necessary. They recognise their strengths, weaknesses and values and those of others and help them to grow. They are *accessible* and colleagues can rely on them when needed. Clinical leaders also possess *informal leadership skills*. They are respected by their colleagues and expected to give compliments while also letting them know when something is not working well. They are supported in the team and will informally lead others by giving advice to colleagues in different situations.

They are mandated by colleagues to say something, for example a compliment, but also if something goes not well. (Healthcare professional with a formal leadership role, FG2)

#### 3.2.5 Visionary

Clinical leaders are *visionary* and *involved* in the organisation they work for. They show an active engagement and actively propagate the vision of the organisation, adopt a loyal attitude and share their viewpoints. They act *purposively*, guard the path to be walked and know very well which direction they want to go. They get things done by the vision they radiate and manage to think along the pre‐set lines within the nursing home. Above all, they want to move forward and have a common goal in mind.

They can think within the lines set out by the nursing home. (Healthcare professional with no formal leadership role, FG5)

#### 3.2.6 Commitment

Clinical leaders fulfil their job with a certain *passion*. They are enthusiastic, dynamic and driven healthcare professionals. They do their job with love, inspiration and have a heart for residents. They have a *positive attitude* and image of their profession. Clinical leaders are *dedicated* to their job, are ambassadors of good care and *spread* this widely. They show a lot of commitment to residents and colleagues and are the healthcare professionals who work ‘with heart and soul’ in the nursing home.

He is a passionate person, always busy, full of enthusiasm and with a smile. (Healthcare professional with no formal leadership role, FG1)

#### 3.2.7 Resilient

Clinical leaders are flexible, *open‐minded*, cope well with changes and put things into perspective when necessary. They *adapt* easily to different and unexpected situations. Clinical leaders do things in their own way and are tolerant of others' differing viewpoints. They do not participate in a negative spiral and turn these negative situations into positive ones. They are flexible, interested in other people's perspectives and behave impartially. They are courageous and creative problem‐solvers. Clinical leaders are *benevolent*, enjoy taking their time and are available to give others attention when needed. They look beyond their own range of tasks and take over extra tasks from colleagues. They are helpful towards residents and colleagues and put others first, although without losing themselves. They are *stress‐resistant* and can cope with the high work pressure that prevails in nursing homes.

They can put things into perspective and remain neutral when things explode. (Healthcare professional with a formal leadership role, FG4)

#### 3.2.8 Responsive

Responsiveness, defined as the quality of having a reaction to something or someone, especially a quick or positive reaction, was found in the participants' perceptions. Clinical leaders are *decisive*, they take initiative, approach tasks carefully and are result‐oriented. Instead of waiting for things to happen, they look ahead and anticipate, dare to tackle situations and solve problems. They share responsibility with the other members of the team. Clinical leaders are *careful*, observant and have an eye for detail, which means that they are reliable and consistent.

They are healthcare professionals who enable things and actions and get the ball rolling when the situation requires it. (Healthcare professional with no formal leadership role, FG4)

## 4 DISCUSSION

We collected and analysed qualitative data on the perception of nursing home healthcare professionals regarding the concept of clinical leadership (research question 1) and the personal characteristics of clinical leaders (research question 2) in the context of nursing homes in Flanders.

Several participants in our study identified informal leadership skills. They said that clinical leadership was practiced by healthcare professionals who did not possess formal authority as leader. This was also found by Boamah (2019) who stated that clinical leadership at the informal level has emerged in nursing. This is a promising situation because Zúñiga et al. (2019) described the added value of informal clinical leaders in nursing homes by their ability to improve the quality of care and reduce hospitalisations. From a grounded theory approach, data saturation in our study was reached at the point in analysing the data when the researchers did not find no more additional data leading to new themes, as described by Birks and Mills (2022) and Olshansky (2015).

An important issue in the literature is that clinical leadership may occur in formal and informal positions. Today, it remains unclear whether the concept of clinical leadership in nursing homes should be linked to both forms of leadership (Fiset et al., 2017; Stanley & Stanley, 2018). Most articles on clinical leadership describe a formal leadership role for nurses working at the middle management level. Strengthening their leading role and supporting them can benefit the work situation (Backman et al., 2018). In nursing homes, the concept of clinical leadership is unknown (Fiset et al., 2017). This is in accordance with the results from this study.

The strength of this study is that participants were practicing experts with a different professional background. This is important because current evidence is limited to the nursing and medical professions (Barnes et al., 2020; Stanley & Stanley, 2018; Zúñiga et al., 2019). The main finding was that informal clinical leaders had an important role in strengthening the quality of multidisciplinary team‐based care. Future research on this topic might address the worldwide challenge to improve the quality of teamwork in healthcare (Busari et al., 2020).

The responses to the second interview question ‘what are the personal characteristics of clinical leaders’ were helpful in answering the second research question. Participants were successful in identifying an informal clinical leader. First, they found it easy to identify a colleague who was a clinical leader or role model for them. This finding is opposed to the finding of Stanley and Stanley (2018). They described that the absence of competency frameworks for clinical leadership in nursing homes made it difficult to recognise clinical leaders (Stanley & Stanley, 2018).

The researchers analysed the characteristics and clustered them into eight themes to define clinical leaders for nursing homes in Flanders. Clinical leaders provide person‐centred care (theme 1). They have strong communication skills (theme 2). They are clinical experts in their field and they are motivated to engage in lifelong learning (theme 3). They are team players and aware of their informal leadership skills (theme 4). They are visionary and loyal to the nursing home's vision (theme 5). They are committed to the resident and the team (theme 6), resilient (theme 7) and responsive (theme 8). The definition that best fits the participants' responses is formulated by Chavez & Yoder in Stanley and Stanley (2018). They described clinical leaders as nurses who provide day‐to‐day care, act as role models, influence, motivate and inspire others with their values and beliefs to improve care, although they have no formal authority. Clinical leaders in nursing homes are showing valuable characteristics to cope with daily hazards, stressful crisis situations and are encouraged to make a positive difference for older residents. Yet, clinical leaders may not be perceived as being superwomen or supermen who are ‘doing it all good’. A heroic representation of clinical leaders holds the risk that no healthcare professional recognises themselves in being a clinical leader. Yet, participants in this study recognised that each healthcare professional already exhibits at least one or more characteristics of clinical leadership.

The first and main theme was *person‐centredness*. This is an interesting finding because this was not the main theme in the clinical leadership literature in hospital settings where the focus lied on the clinical expert role. This is not a surprising finding given the differences in context between hospitals and nursing homes. Closely related to this first theme was the second theme, *effective communicator*. The importance of communication skills in quality care cannot be overestimated. Even the first definition of clinical leadership included communication (Harper, 1995) and communication also appears regularly as a core competency in the hospital setting (Ashour et al., 2022; Mrayyan et al., 2023; Stanley & Stanley, 2018). The third theme was ‘*clinical expert*’, a theme that was also found in numerous studies and described as clinical competency and being a good clinical practitioner (Ashour et al., 2022; Mianda & Voce, 2018; Stanley & Stanley, 2018). Similar to the findings in this study, Stanley and Stanley (2018) named focusing on excellence and delivering quality care as important characteristics. The fourth theme, *team‐based worker*, appeared to be an important competency of clinical leadership and has been mentioned several times in the hospital care literature (Mianda & Voce, 2018; Mrayyan et al., 2023; Stanley & Stanley, 2018). The fifth theme, being a *visionary* leader, was important for participants. Different researchers already confirmed this finding, but Stanley and Stanley (2018) did not agree. They found that the characteristic visionary was rarely identified as a clinical leadership attribute. The sixth theme, *committed*, has not yet been directly linked to clinical leadership. Some characteristics, such as motivated and enthusiastic, were already identified in previous research (Stanley & Stanley, 2018). The seventh theme, *resilient*, was called flexibility or adaptability in other studies, and researchers follow the statement resilience is seen as an important characteristic of clinical leaders going beyond flexibility (Mrayyan et al., 2023; Stanley & Stanley, 2018). The final theme, *responsive*, was new. The authors could not find the same word in the literature, they found characteristics linked to responsiveness such as problem‐solving, goal setting, decision‐making and working conscientiously (Enghiad et al., 2022; Stanley & Stanley, 2018). It is noticeable that several characteristics overlap. The characteristic person‐centred care is key in nursing homes and necessary to answer the holistic needs of residents. For example, healthcare professionals need clinical expertise to collaboratively manage highest complexity and statistically significant risk in the support for older persons to deliver person‐centred care.

As described above, and in contrast to the clinical leadership concept in hospitals, person‐centredness (theme 1) and team‐based worker (theme 2) got a central place in the figure (Figure 1), surrounded by all the other personal characteristics of a clinical leader. Having the characteristics to provide person‐centred care is a condition to be able to act as a clinical leader in the context of nursing homes. This figure is similar to the flower figure of the CanMEDS (Canadian Medical Education Directions for Specialists) competency framework, a framework to describe competencies for physicians and consisting of seven roles: medical expert, communicator, collaborator, leader, health advocate, scholar and professional (Frank et al., 2010). This brings us to some points of debate.

First, this study aimed to identify the personal characteristics of a clinical leader. The question is if these characteristics can be trained and assessed, or if they belong to the personality of a healthcare professional. The researchers saw different dimensions in the results, some belonging to attitudes (e.g. resilience), some to knowledge (e.g. clinical expertise) and some to skills (e.g. communication skills). Therefore, the authors want to emphasise the importance of developing and validating a competency framework in the context of nursing homes. Competencies are defined as the ability(ies) of a person to integrate knowledge, skills and attitudes in their performance of tasks in a given context. Competencies are durable, trainable and, through the expression of behaviours, measurable (Englander et al., 2017). The results in this study, together with the existing competency frameworks in healthcare, can be used as the basis to develop these competencies. Some themes already clearly refer to domains of the existing competency frameworks such as expert (theme 3), communicator (theme 2) and collaborator (theme 4) from the CanMEDS competency framework. Other characteristics can be mapped to existing competencies and competency domains in the different frameworks.

Secondly, once the competencies are outlined, specific competency‐based training courses can be designed to develop competencies during undergraduate, postgraduate and continuing education (Lucey et al., 2018). High‐quality care and quality of life for older people requires engagement in the professional development of staff, addressing knowledge, skills and leadership development (Fitzpatrick et al., 2023). Furthermore, monitoring and assessing competency development using an ePortfolio is another challenge to be investigated in the future (Janssens et al., 2022).

Finally, this study provides an overview of the personal characteristics of clinical leaders in nursing homes. Because of the numerous challenges in nursing homes, future research might be valuable to study how personal characteristics of clinical leaders in nursing homes can be appropriately identified, supported, rewarded and developed (Enghiad et al., 2022). When clinical leaders are appropriately identified (e.g. based on a self‐awareness tool), it might be enabled that healthcare professionals reflect on their clinical leadership characteristics, awakening their professional development and growth (e.g. with the support of the formal leader), which might benefit quality of care in nursing homes (Ashour et al., 2022). Also, awareness of the working environment (e.g. adequately staffed, have adequate resources, supportive formal leaders, etc.) is essential to support the development of healthcare professionals in nursing homes (Potrebny et al., 2022). Investigating the impact of clinical leadership on the enhancement of quality measures (e.g. job satisfaction) and health outcomes (e.g. morbidity, well‐being of residents) is another interesting topic for the future (Mrayyan et al., 2023).

This study is valuable because to our knowledge it is the first study investigating the concept of clinical leadership and personal characteristics of clinical leaders in the setting of nursing homes. Another strength is that participants had different professional backgrounds and were part of the multidisciplinary team. Some limitations of this study need to be considered. The most important limitation was that the participating group represented only a small percentage of the entire healthcare professionals' cohorts in Flanders. A broad group of healthcare professionals (healthcare professionals with formal and no formal leadership roles) was reached to participate in the focus groups. Healthcare professionals in formal leadership roles were included because formal leaders in Flemish nursing homes also often partially engage in resident care (e.g. in case of staff shortage, etc.). Next, the sample consisted of 61% healthcare professionals in no formal leadership role and 39% healthcare professionals in a formal leadership role. It is important to note that 89.1% of the participants were employed within a nursing home with a bed capacity of more than 150 beds and the last two focus groups were organised in the same nursing home with more than 150 beds. The organisational culture prevailing in this organisation may possibly have influenced the results, making them less applicable nursing homes with a lower bed capacity. Because of the small sample and specific context of nursing care in Flanders, the generalisability of the results may be limited.

## 5 CONCLUSION

The results of this study suggest that clinical leadership in nursing homes could be defined as passionate healthcare professionals providing person‐centred care with strong communication skills. They are clinical experts in their field and motivated to engage in lifelong learning. They are team players with informal leadership skills. They are visionary, committed, resilient and responsive. They are identified as informal clinical leaders strengthening the multidisciplinary team‐based care. The current challenge is to explore the relationship between the identified characteristics (person‐centred, effective communicator, clinical expert, team‐based worker, visionary, committed, resilient and responsive) and competency‐based approaches in healthcare practice and education. Future research should focus on the development of competencies, training courses, monitoring and assessment methods to improve the evidence of clinical leadership in nursing homes and to support the implementation in practice.

## 6 IMPLICATIONS FOR PRACTICE

- Informal clinical leaders play an important role in strengthening the quality of multidisciplinary team‐based care for residents in nursing homes.
- The defined characteristics, together with the existing competency frameworks in healthcare can be used as the basis to develop clinical leadership competencies in nursing homes.
- Awareness of the definition and the main characteristics of clinical leadership is necessary to facilitate the identification, support and development of healthcare professionals in nursing homes.

## AUTHOR CONTRIBUTIONS

SN: conceptualisation, methodology, data collection, data analysis, writing – original draft preparation, review and editing. NDR: conceptualisation, methodology. JA: conceptualisation, methodology. PDV: conceptualisation, methodology, data collection, data analysis, review and editing. ME: writing – original draft preparation, review and editing. EC: conceptualisation, methodology, data analysis, writing – original draft preparation, review and editing.

## 7 FUNDING INFORMATION

The research was conducted as part of the employment of the authors within Artevelde University of Applied Sciences and without specific external funding.

## CONFLICT OF INTEREST STATEMENT

The authors declares that there is no conflict of interest regarding the publication of this paper.

## Data Availability

The data that support the findings of this study are available on request from the corresponding author. The data are not publicly available due to privacy or ethical restrictions.
